# 

**Published:** 1960-07

**Authors:** 


					*// O 53
/r/^gsy
THE
MEDICAL JOURNAL
SUffFlUS
OF THE
SOUTH
I duplTcate
Incorporating
The Bristol Medico-Chirurgical Journal
July i960
L- 75 (iii). No. 277 Price 4/-

				

